# Cloning and Characterization of a 7 Transmembrane Receptor from the Adherent Cells of Chicken Peripheral Blood Mononuclear Cells

**DOI:** 10.1371/journal.pone.0086880

**Published:** 2014-01-22

**Authors:** Yu San Chen, Hsing Chieh Wu, Jui Hung Shien, Hua Hsien Chiu, Long Huw Lee

**Affiliations:** 1 Department of Veterinary Medicine, College of Veterinary Medicine, National Chung Hsing University, Taichung, Taiwan; 2 Department of Biotechnology, College of Environmental and Life Science, Fooyin University, Kaohsing, Taiwan; Russian Academy of Sciences, Institute for Biological Instrumentation, Russian Federation

## Abstract

A cDNA encoding a 7 transmembrane (7TM) receptor gene from the adherent cells of chicken peripheral blood mononuclear cells (PBMC) was cloned and characterized. The open reading frame of the chicken-7TM (Ch-7TM) receptor gene was 1008 nucleotides long, encoding a protein of 335 amino acid residues with a molecular mass of approximately 37.1 kDa. Hydrophobic stretches indicated the presence of 7 TM domains. Moreover, the complete nucleotide sequences encoding 7TM of duck (Du-7TM) and goose (Go-7TM), corresponding to the open reading frame of Ch-7TM, were determined. Each of the Du- and Go-7TM encoding regions comprised 990 nucleotides, representing an 18-nucleotide deletion in alignment with the Ch-7TM encoding region, resulting in a 6-amino-acid deletion at the 3′-end. No signal peptides were predicted. Six phosphorylation sites were predicted and conserved for all three 7TMs. The proteins of the three 7TMs were similar, with 11 conserved cysteine residues. No glycosylation sites could be predicted. The results of the pairwise comparisons indicated that the Ch-7TM encoding region and Ch-7TM protein were the least similar to those of Du- and Go-7TMs. These results were in accordance with those of the phylogenetic analysis, which indicated that the Du- and Go-7TM encoding regions clustered, but were separated from the Ch-7TM encoding region. Monoclonal antibody B28D5 was prepared from spleens of mice immunized with the bacterially expressed N-terminal (55 amino acid residues) region of the Ch-7TM protein for further use. Double staining with B28D5 and KUL01 suggested that Ch-7TM was expressed in subsets of the adherent cells, among which a subset that was recognized with both antibodies was likely of monocyte and macrophage lineage. However, the fluorescence intensities of B28D5 and, particularly, KUL01 decreased after the adherent cells were incubated for additional 48 h.

## Introduction

It is thought that the development of mononuclear phagocytes in birds is the same as that in mammals [1]. Macrophages originate from bone marrow stem cells by differentiating into monoblasts, promonocytes, and monocytes [2]. The general morphology and distribution of the different subpopulations of mononuclear phagocytes in birds and mammals are considerably similar. Monocytes migrate from the peripheral blood to the tissues and differentiate into macrophages [3].

Macrophages play crucial roles in various immune responses [2]. On the outermost surface of the cells, the membrane-associated receptor binds to its ligand counterpart, and displays an array of biological activities. The 7 transmembrane receptors (7TM) are the largest and most ubiquitous family of membrane receptors in multicellular invertebrates [4] and vertebrates [5]. Their ligands are highly diverse, including hormones, ions, chemokines, neurotransmitters, and light photons. Based on their sequence similarities, 7TM receptors are grouped into 3 major families: A, B, and C [6]. Many 7TM receptors signal by activating heterotrimeric G proteins after binding to their ligands, resulting in the phosphorylation of different substrates. Two 7TM receptors identified in chicken leukocytes have been described. One receptor was identified in chicken heterophils [7] and the other was identified in a chicken HD11 macrophage cell line exposed to a *Salmonella*-derived endotoxin [8]. Other 7TM sequences, which were predicted to be the MAS-related G protein-coupled receptor (GPCR) gene and its nucleotide sequences, have been deposited in GenBank.

In this study, we cloned and characterized the entire length of a cDNA encoding a 7TM receptor from the adherent cells prepared from chicken peripheral blood mononuclear cell (PBMC). The open reading frame (ORF) of the chicken-7TM receptor gene was 1008 nucleotides long, encoding a 335-amino acid protein. Double immunofluorescence staining of the adherent cells cultured for 1 h and 48 h of chicken PBMC using monoclonal antibodies (MAb) B28D5 and KUL01. B28D5 and KUL01 recognize the N-terminal part of the Ch-7TM protein and chicken monocytes and macrophages, respectively. It indicated that subsets of the Ch-7TM-expressing adherent cells, which were recognized with both antibodies, were likely of mononcyte and macrophage lineage. In addition to the Ch-7TM, we also cloned, sequenced, and characterized encoding regions of Pekin-duck (*Anas platyrhynchos domestica*) (Du)-7TM (Du-7TM) and the White–Roman-goose (*Coscoroba coscoroba*) (Go)-7TM (Go-7TM) of the adherent cells of PBMC to further analyze the evolution of these protein-encoding genes. The results indicated that the 7TM encoding regions and the deduced amino acids of the duck and goose clustered together, and were more similar to the sequences of the mallard (*Anas platyrhynchos*) (accession number: XM_005028391) than to the sequences of the chicken. Similarly to the 7TM gene sequences of avian species deposited in GenBank (accession number: XM_005028391), which have been predicted to be the MAS-related GPCR protein gene, the Ch-, Du-, and Go-7TM gene sequences cloned in this study were predicted to be the MAS-related GPCR protein sequences belonging to Family A (the rhodopsin-related family GPCRs) of the 7TMs [6].

## Materials and Methods

### Animals

Specific pathogen-free (SPF) Leghorn chickens were obtained from SPF flock managed by the Animal Health Research Institute, Council of Agriculture, Taiwan. BALB/c mice were purchased form the National Laboratory Animal Center, Nang- gang, Taipei, Taiwan. All experimental protocols for animal trials were approved by the Animal Care and Uses Committee, National Chung Hsing University (NCHU) (permit number: 99-88). The experiments were conducted based on the Ethical Rules and Law of the NCHU.

### Cell culture

Chicken adherent cells were prepared from the blood of SPF Leghorn chickens [9]. Leukocyte fractions were obtained and cultured in RPMI 1640 (Gibco/Life Technologies, Carlsbad, USA) supplemented with 10% chicken serum (Gibco) and 100 U/mL of penicillin and streptomycin (Life Technologies) in 6-well cell culture plates for 1 h at 37°C. After removing non-adherent cells, the adherent cells were scraped using a cell scraper and resuspended with the same medium. The cell density was adjusted to a concentration of 1×10^5^ cells/mL. The cells were cultured in 24-well tissue culture plates, with or without coverslips, for an additional 48 h (adherent cells/48 h). Part of the leukocyte fraction was cultured in 24-well tissue culture plates with coverslips for 1 h at 37°C. After removing non-adherent cells, the adherent cells (adherent cells/1 h) were used for further study. HeLa cells were grown in Dulbecco modified Eagle medium (DMEM) supplemented with 10% fetal calf serum (Gibco) and antibiotics, as previously mentioned, to construct stable cell lines. The medium used to maintain stable cell lines also contained 200 µg/mL of G418 (Life Technologies). For immunostaining assays, stable cell lines were grown on coverslips.

### cDNA library and nucleotide sequence analysis

The total RNA was extracted from the adherent cells/48 h prepared as previously described, using Trizol reagent (Life Technologies) according to the protocol of the manufacturer. cDNA prepared from poly (A)-rich RNA with SuperScript™ III reverse transcriptase (Invitrogen) was undirectionally cloned into pUC18, and the recombinant plasmids were transformed into *Escherichia coli* DH5α. The white colonies were randomly selected. The plasmids were amplified in *E. coli*, and the insert was sequenced. The obtained nucleotide sequences were analyzed by translating them into amino acid sequences. After the predicted amino acid sequence having more than one hydrophobic transmembrane domain was identified, RACE was used as described by the manufacturer (Life Technologies) to obtain the full-length cDNA of these clones until a 7TM receptor gene (Ch-7TM) was attained. The ORF encoding the 7TM domains was predicted using the TMHMM 2.0 (http://www.cbs.dtu.dk/services/TMHMM/) server. Signal peptides were predicted using the SignalIP 4.1 (http://www.cbs.dtu.dk/services/SignalIP/) and Signal-3L (http://www.csbio.sjtu.edu.cn/bioinf/Signal-3L/) severs. N-linked glycosylation sites were predicted according to the presence of NXS/T motifs, and phosphorylation sites were predicted using the NetNGlyc 1.0 (http://www.cbs.dtu.dk/services/NetNGlyc/) and NetPho 2.0 (http://www.cbs.dtu.dk/services/NetPhos/) servers. Amino acid sequences were aligned using the ClustalW2 algorithm (http://www.ebi.ac.uk/Tools/msa/clustalw2/) and adjusted manually. To assess the phylogenetic relationships of Ch-7TM among avian species, we cloned, sequenced, and characterized the encoding regions of Du-7TM and Go-7TM that corresponded to the ORF of Ch-7TM. The total RNA from the adherent cells of duck and goose PBMC, prepared as described for chicken adherent cells, was extracted and reversibly transcribed into cDNA using oligo dT as primer and with SuperScript™ III reverse transcriptase (Invitrogen). The 7TM encoding region of each avian species was then amplified by a DNA polymerase *Pfu* (Fermentas, Burlington, Ontario) in polymerase chain reaction (PCR) using the cDNA products of each avian species as the template [9]. Based on the Ch-7TM sequences obtained in this study, the primers were designed as follows: the forward primer (5′-GCAGCAGTGGTTTCACAATG-3′) corresponded to nucleotides 239–258, numbered according to the cDNA sequence of the Ch-7TM gene, and the reverse primer (5′-GACTCTCCTACCCAGCAACA-3′) was complementary to nucleotides 1439–1458, numbered according to the cDNA sequence of the Ch-7TM gene. Both primers were used to amplify each 7TM encoding region of the 3 avian species. The amplified products were directly used for sequencing in both directions. To validate the identity of the sequence, sequencing was carried out on the PCR products from at least 3 independent experiments. The sequences were analyzed using the ABI Prism 337 sequence analyzer (Perkin-Elmer, Waltham, USA). The nucleotide sequences from the 7TM encoding regions of all 3 avian species were aligned to create a phylogenetic tree for relationship studies, using the clustal method of the DNASTAR software (DNASTAR Inc, Madison, WI, U.S.A.).

### Expression and purification of the N-terminal region of the Ch-7TM protein

Antigens used for the production and characterization of MAbs were synthesized in *E. coli* BL21 (DE3) as described previously [10]. Briefly, the ORF corresponding to the predicted extracellular region (Ch-7TMN) of the Ch-7TM gene (Met^1^-Glu^55^) was amplified and cloned into a pET28a vector (Novagen, Darmstadt, Germany) to generate pET28a-Ch-7TMN, which was subsequently transformed into *E. coli* BL21 (DE3) (Novagen). After induction with 0.1 mM of IPTG for 4 h, the soluble proteins were purified using a His-Bind resin column (Novagen) according to the protocol of the manufacturer. The size and presence of the recombinant Ch-7TMN (rCh-7TMN) was verified using standard western blot methods with an anti-His antibody (AbD Serotec, Kidlington, UK) as the primary antibody, and a goat anti-mouse conjugated-AP antibody as the secondary antibody.

### Production and characterization of MAbs

BALB/c mice were immunized intraperitoneally with 25 µg of rCh-7TMN in 0.2 mL of Freund complete adjuvant, and boosted twice with the same amount of rCh-7TMN in Freund incomplete adjuvant at a 2-wk interval. Six weeks after the initial vaccination and 4 d before mice were sacrificed to prepare hybridoma, a final boost was carried out in the same route using the same amount of antigens in 0.1 mL of phosphate buffered saline (PBS).

MAbs were prepared using previously described techniques [11]. Briefly, splenocytes from the mice immunized with rCh-7TMN antigens were fused with NS1 myeloma cells. Hybridoma cell lines that secrete antibodies that recognize Ch-7TM proteins were screened and subcloned at least 3 times, using a limited dilution method, and the ascitic fluids were prepared using the cloned hybridoma in pristane-primed mice.

Hybridoma culture supernatants were screened for antibodies against rCh-7TMN in an indirect enzyme-linked immunosorbent assay (ELISA) as described previously [12], except rCh-7TMN antigens were used for plate coating. Ch-7TM-specific MAbs were further confirmed by conducting immunofluorescent antibody staining of the stable cell lines HeLa-eGFP and HeLa-Ch-7TM/eGFP. The immunoglobulin (Ig) class of each MAb present in the individual ascitic fluids of the mice was determined by performing an ELISA using a Zymed MAb kit.

### Establishment of stable cell lines

The entire ORF of eGFP was amplified from a recombinant fowl pox virus [13] by conducting a PCR and cloned into pcDNA3.1 (Invitrogen) using the *EcoRI* and *XhoI* cloning sites in plasmid multiple cloning sites (MCS) to generate pcDNA3.1/eGFP. To generate pcDNA3.1/Ch-7TM-(Gly_4_ Ser)_3_, the entire ORF of Ch-7TM was amplified from the previously prepared total RNA of the adherent cells/48 h by using a reverse transcription-PCR. The 5′-terminal and 3′-terminal primers contained *Bam HI* and *EcoRI* restriction sites, respectively. The amplified cDNA was then cloned into pcDNA 3.1, using the *Bam HI* and *EcoRI* sites from the MCS. Finally, the amplified ORF of eGFP was subcloned into pcDNA3.1/Ch-7TM-(Gly_4_ Ser)_3_, using the *EcoRI* and *Xho I* cloning sites to generate pcDNA3.1/Ch-7TM/eGFP. The structures of the resulting plasmids were confirmed directly by conducting DNA sequencing using an ABI 377 automated sequencer. The stable cell lines HeLa-eGFP and HeLa-Ch-7TM/eGFP were established using the G418 selection of HeLa cells, and were subsequently transfected using the Effectene transfection reagent (Qiagen) as described previously [14]. HeLa-eGFP synthesized an eGFP protein, whereas HeLa-Ch-7TM/eGFP produced a fusion protein containing Ch-7TM and eGFP linked by a linker sequence (Gly_4_ Ser)_3_.

### Immunostaining

After subcloning, clones with a positive reaction to rCh-7TMN, screened using an ELISA, were selected for further confirmation through immunostaining. The stable cell lines HeLa-eGFP and HeLa-Ch-7TM/eGFP were grown on coverslips, fixed in cold acetone for 20 min, and incubated with the culture supernatant of the positive clones, and, subsequently, a secondary antibody conjugate (PE-conjugated rabbit anti-mouse IgG; Jackson ImmunoResearch Laboratories, West Grove, PA, USA). One clone (B28D5) was selected for additional analyses. To determine whether the adherent cells of chicken PBMC synthenized Ch-7TM protein, the cells grown on coverslips were fixed in cold acetone for 20 min and visualized with PE-conjugated KUL01 (1∶10) (Southern Biotech, Birmingham, AL. USA). Afterward, they were double-stained with Ch-7TM, using MAb B28D5 and Alexa flour 488-conjugated rabbit anti-mouse IgG (Invitrogen) as the primary and secondary antibodies, respectively. Blue 4′,6-diamidino-2-phenylindole (DAPI) was used for nuclear counterstaining. Images of immunostaining were captured using a confocal microscope (Zeiss, Oberkochen, Germany). The nature of the proteins recognized by MAb B28D5 was confirmed using a SuperSignal® West Femto Maximum Sensitivity Substrate kit (Thermo Scientific, Pierce Biotechnology, Rock Ford, IL) according to the instruction manual.

## Results and Discussion

### Cloning and sequence analysis of the Ch-7TM gene and the 7TM encoding regions of 3 avian species

**Figure 1:**
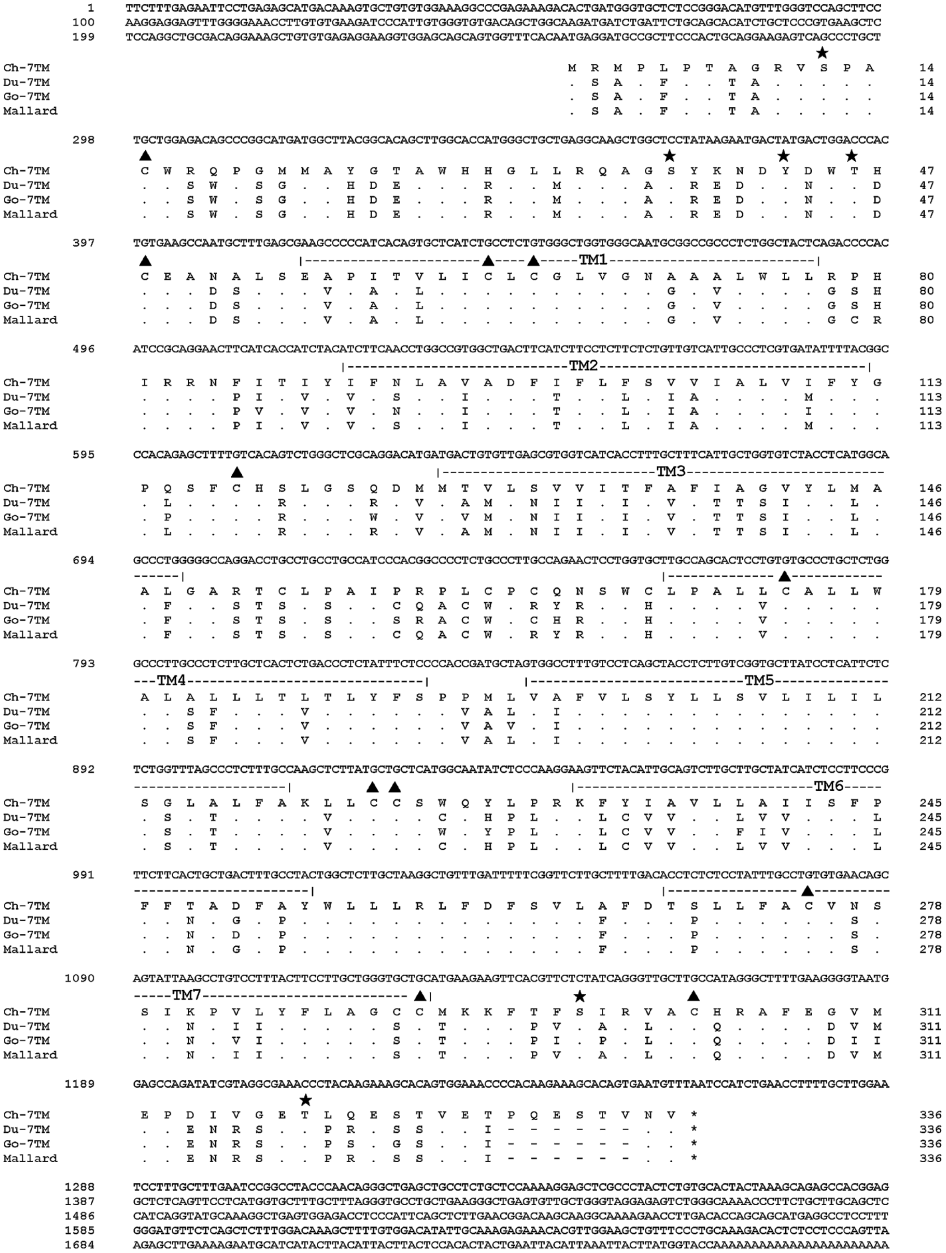
Nucleotide sequence of the Ch-7TM gene, and alignment of the Ch-, Du-, and Go-7TM proteins. Complete nucleotide sequence of the Ch-7TM gene was 1761 bp long. The Ch-7TM consisted of one long open reading frame with 1008 nucleotides, which started with ATG (at residues 256 to 258) and terminated at nucleotides 1261 to 1263. The non-coding regions at the 5′- and 3′-ends were 255 and 496 nucleotides long, respectively. The 7TM amino acid sequences of the 3 avian species were aligned. Amino acid residues of the Ch-7TM are shown as a single-letter code on the top of the amino acid alignment. Residues that were identical to the amino acid sequence of the Ch-7TM are indicated by dots (•). Gaps in the sequences are indicated by dashes (-). Positions that are predicted to be phosphorylated are indicated as (★). Positions of the cysteine residues of the three 7TMs are indicated as (▴). The predicted transmembrane domains (TM1-TM7), relative to Ch-7TM, are marked by a line spanning the region.

A total of approximately 3700 individual white colonies, which potentially contained inserts, were obtained using the prepared cDNA library. Sequencing 537 clones yielded 32 clones with partial sequences encoding a probable 7TM receptor. Of the 32 clones, one clone (Ch-7TM) encoded a 7TM receptor. The complete nucleotide sequence of the Ch-7TM cDNA, along with part of the poly (A) tail, is shown in Fig. 1, together with the deduced amino acid sequence (accession number: KF555642). The Ch-7TM gene contained one long ORF comprising 1008 nucleotides; it started with an ATG (at residues 256 to 258) and terminated at nucleotides 1261 to 1263, encoding a protein of 335 amino acid residues with a molecular mass of approximately 37.1 kDa. The non-coding regions at the 5′- and 3′- ends were 255 and 496 nucleotides long, respectively. The hydropathy diagram [15] of Ch-7TM revealed a pattern that was typical of GPCRs. Hydrophobic stretches indicated the presence of 7TM domains. Based on the Ch-7TM sequence, the complete nucleotide sequences of Du-7TM- and Go-7TM-encoding regions were determined, and were submitted to GenBank. The encoding regions of Du-7TM and Go-7TM (accession numbers: KF555643 and KF555644, respectively) were 990 bp, which is the same size as that of the mallard (accession number: XM_005028391), but were different from that of the Ch-7TM encoding region, which was 1008 bp. Each of the Du-7TM and Go-7TM encoding regions displayed an 18-nucleotide deletion in alignment with the Ch-7TM encoding region, resulting in 6-amino acid deletions at the 3′ end (Fig. 1). Each Du-7TM and Go-7TM encoding region encoded a 7TM protein containing 330 amino acids, which is the same size as that of the mallard (accession number: XM_005028391). The Ch-7TM encoding region encoded a 7TM protein that contains 335 amino acids. The Ch-7TM encoding region showed a 100% identity in alignment with part of the predicted mRNA nucleotide sequence of chicken (the LOC101752158 gene), which is 2981 nucleotides in full length (accession number: XM_004950707). In addition, the 3′ non-coding region of Ch-7TM showed a low percentage of identity with the chicken LOC101752158 gene, and Ch-7TM did not contain the additional sequences downstream from the 3′ end that were predicted for the chicken LOC101752158 gene. Because the 7TMs of all 3 avian species were similar to that of the mallard in size and sequence identity, Ch-, Du-, and Go-7TM proteins were classified into the MAS-related G protein belonging to Family A (the rhodopsin-related family GPCRs) of the 7TM [6].

To analyze the amino acid sequences, Ch-, Du-, and Go-7TM proteins were aligned (Fig. 1). The ORF did not contain an archetypal, cleavable signal peptide or signal sequence, and likely underwent secretion to the cell membrane through a mechanism that exposed the amino terminus on the exterior surface of the membrane [16]. As with other GPCRs, Ch-7TM may undergo some posttranslational modifications [17]. Phosphorylation sites at 3 serine (Ser^12, 38, 298^), one tyrosine (Tyr^43^), and 2 threonine (Thr^46, 319^) residues were predicted, and were conserved for all three 7TMs. The proteins of the three 7TMs were similar, with 11 conserved cysteine residues. No glycosylation sites could be predicted.

### Sequence comparisons and phylogenetic analysis of Ch-7TM, Du-7TM, and Go-7TM encoding genes and their deduced proteins

**Figure 2:**
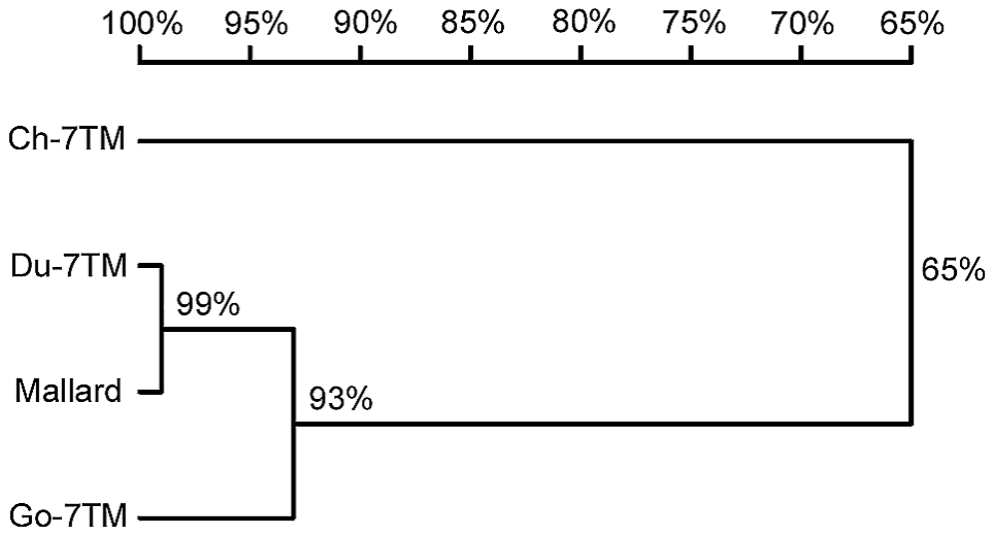
Phylogenetic relationships of the Ch-, Du-, and Go-7TM encoding regions. It was constructed by using the Clustal program of the DNASTAR software package. The length of the horizontal line is proportional to the minimum number of nucleotide differences.

Pairwise sequence comparisons were conducted to examine the degree of sequence identity of the Ch-7TM-, Du-7TM-, and Go-7TM-encoding regions and their 7TM proteins. The results are shown in Table 1. The sequence identities of the 7TM-encoding regions ranged from 75% to 99%, whereas those of the 7TM protein ranged from 64% to 99%. The 7TM encoding regions and the 7TM proteins of Pekin ducks and mallards displayed the greatest identity. The Ch-7TM encoding region and Ch-7TM protein displayed the least identity, whereas those of the other three 7TMs displayed no more than 76% or 65% identity. The results were in accordance with those of phylogenetic analysis, which proved that the Du-7TM and Go-7TM encoding regions clustered, but separated from the Ch-7TM encoding region (Fig 2), suggesting that the 7TM encoding regions of waterfowl (ducks and geese) are more similar to one another than to the 7TM encoding regions of the land-based bird (chicken) regarding evolution. In addition, a total of 7 nucloetide divergences (Table 2) between the 7 TM encoding regions of Pekin-Duck and mallard were observed. Five of 7 nucleotide subtitutions occurred at the third position of codons, resulting in only two amino acid changes at residues 79 (C to S) and 80 (R to H). These results suggested that natural polymorphisms have occurred in 7 TM within the Pekin duck breed during the evolution.

**Table 1:** Percentage identities of the homologous Ch-, Du-, and Go-7TM- encoding regions and the encoded proteins as demonstrated by pairwise comparisons of the homologous protein encoding regions and the deduced proteins.

	Ch-7TM	Du-7TM	Go-7TM	Mallard
Ch-7TM		76	75	76
Du-7TM	64		95	99
Go-7TM	65	93		95
Mallard	64	99	92	

Numbers in the bottom left of the table showed amino acid identities, those in the top right indicated nucleotide identities. On the bases of the pairwise comparison, the number indicated the percentage identity of the 7-TM-encoding regions or the encoded proteins, respectively.

**Table 2:** Nucleotide and amino acid variations of the encoding region of Du-7TM and mallard.

Encoding region	Nucleotide at position							Amino acid at position	
	9^b^	236^a^	239^a^	261^b^	444^b^	546^b^	597^b^	79	80
Mallard	A	G	G	C	T	C	T	C	R
Du-7TM	G	C	A	T	C	T	C	S	H

a Change of nucleotide at the second position of codon between the Du-7TM and mallard resulted in two amino acid changes at residues 79 and 80.

b Change of nucleotide at the third position of codon between the Du-7TM and mallard led to silent results.

### Production and characterization of Ch-7TM MAbs

**Figure 3:**
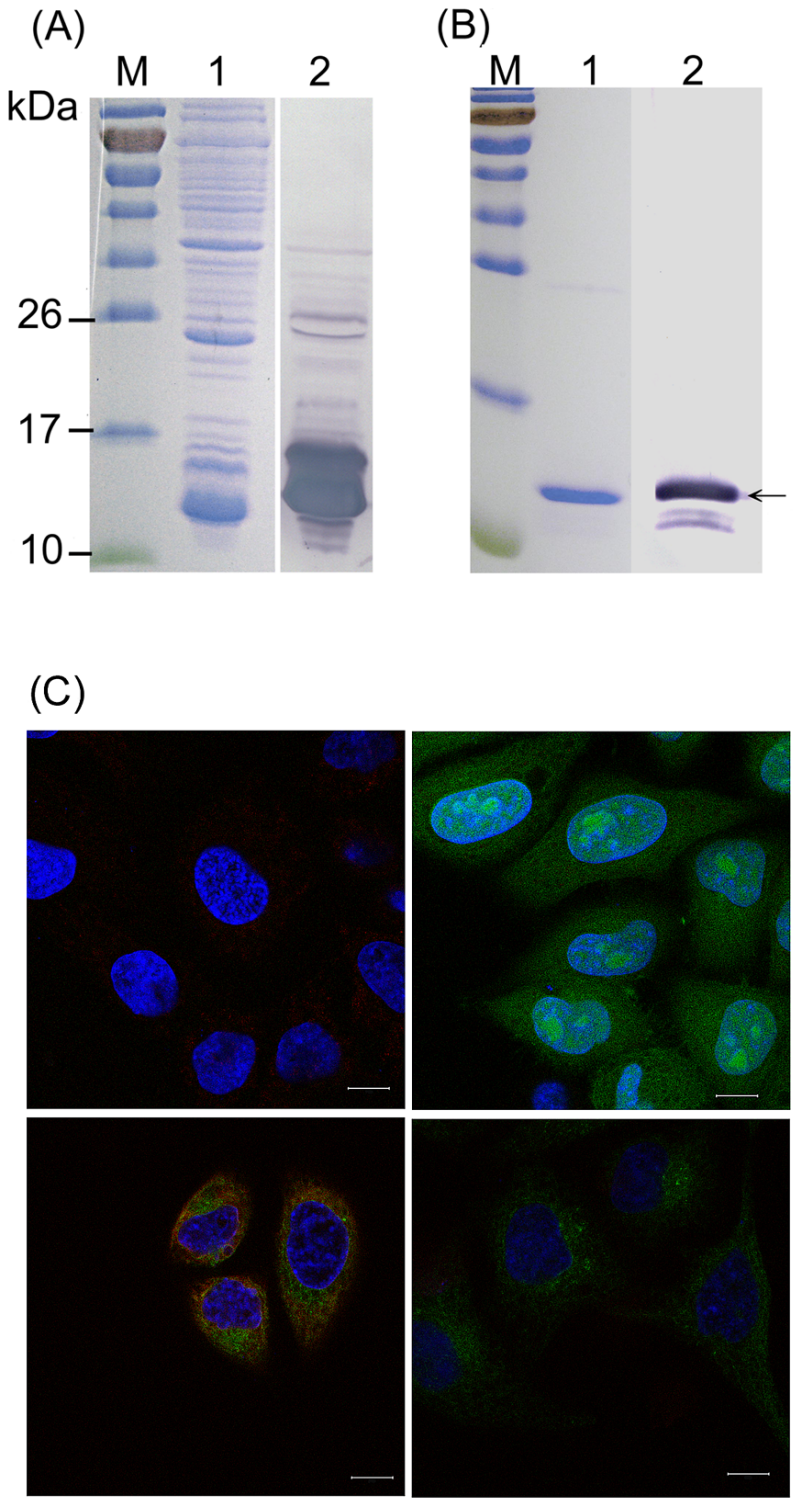
Preparation of monoclonal antibodies against Ch-7TM proteins. (A) The recombinant extracellular part at the N-terminal region of the Ch-7TM protein was expressed in *E. coli* BL21 (DE3) as a fusion protein (rCh-7TMN). Protein expression was induced with IPTG after an additional 3 h, starting 2 h after an OD_600_ of 0.6 was obtained. Entire bacterial cells were separated on SDS-PAGE and stained with Coomassie blue (Lane 1), or the expressed rCh-7TMN protein was probed with anti-His monoclonal antibody (Novagen) using western blotting (Lane 2). Prestained protein markers (Fermentas) (M) in kDa are indicated on the left. (B) From lane 1, bacterial cells were sonicated. After centrifugation, proteins as described for (A) were purified from the supernatant fractions using His-Bind resin. After SDS-PAGE was performed, gels were stained with Coomassie blue (Lane 1), or rCh-7TMN was detected using western blotting (Lane 2) as described for (A). Molecular weight markers (M) are indicated as they are for (A). The purified (Lane 1) and identified (Lane 2) rCh-7TMNs were indicated by arrow. (C) Recognition of the Ch-7TM protein by MAb B28D5 was achieved using immunofluorescent double-staining. Cells were grown on coverslips and fixed in cold acetone for 20 min. Permeabilized HeLa cells (left top); the stable cell line E5E8, which synthesized the Ch-7TM/eGFP fusion protein (left bottom); and the stable cell line D5, which synthesized the eGFP protein (right top), were incubated with the culture supernatant of the positive clone B28D5 and, subsequently, a secondary antibody conjugate (PE-conjugated rabbit anti-mouse IgG). The permeabilized stable cell line E5E8 (right bottom) was incubated with the preimmune serum of mice as the primary antibody. DAPI (blue) was used for nuclear counterstaining. The immunostaining image was captured using a confocal microscope (Zeiss, Oberkochen, Germany). Bar: 10 µm.

To test if the adherent cells of chicken PBMC expressed Ch-7TM protein, MAbs recognizing Ch-7TM were produced and characterized. *E. coli* cells containing the pET28a-Ch-7TMN construct were induced, and the entire cell lysate was examined by sodium dodecyl sulfate-polyacrylamide gel electrophoresis (SDS-PAGE). The result showed that a protein band of approximately 11.55 kDa, which is consistent with the molecular size of the expected fusion protein including the extracellular part (6.38 kDa) and vector (5.17 kDa), was expressed (Fig. 3A, Lane 1). This protein was further identified using the anti-His monoclonal antibody as the primary antibody in western blot analysis (Fig. 3A, lane 2). The soluble fusion protein was purified using His-Bind resin (Novagen). Proteins in a fraction were eluted using binding buffer B-100 mM imidazole, producing a single major band with an estimated molecular size of 11.5 kDa (Fig. 3B, lane 1). The purified protein was further determined using an anti-His antibody (Fig. 3B, lane 2); the result suggested that the expressed and purified protein was the rCh-7TMN, an extracellular part of the Ch-7TM.

A total of 87 hybridomas were screened from the spleen cells of mice immunized with purified rCh-7TMN. To confirm the identities of the produced MAbs that specifically recognized Ch-7TM proteins, stable cell lines E5E8 (Hela-Ch-7TM/eGFP), which synthesized Ch-7TM/eGFP fusion proteins, and D5 (Hela-eGFP), which synthesized eGFP proteins, were constructed to further screen the MAbs. Five of these hybridomas were randomly selected to test the secreted MAbs that recognized the Ch-7TM synthesized in the E5E8 stable cell line, using confocal microscopy. The results demonstrated that all 5 clones secreted MAbs recognizing Ch-7TM in the stable cell line E5E8. A representative clone (B28D5) stained the permeabilized stable cell line (Fig. 3C). MAb B28D5 recognized Ch-7TM in the E5E8 stable cell line (Fig. 3C, left bottom), but not in the D5 stable cell line (Fig. 3C, right top) or the HeLa cell line (Fig. 3C, left top). No binding reaction was obtained when the preimmune serum of mice was used (Fig. 3C, right bottom). Thus, a B28D5 hybridoma was subcloned through limited dilution, and was selected to produce MAbs in mice. The ascitic fluids were characterized. The isotype of MAb B28D5 was IgG1, as determined using the Zymed MAb kit. Characterization of the remaining 82 hybridomas and their MAbs is currently ongoing in our laboratory.

### Ch-7TM expression in the adherent cells of chicken PBMC

**Figure 4:**
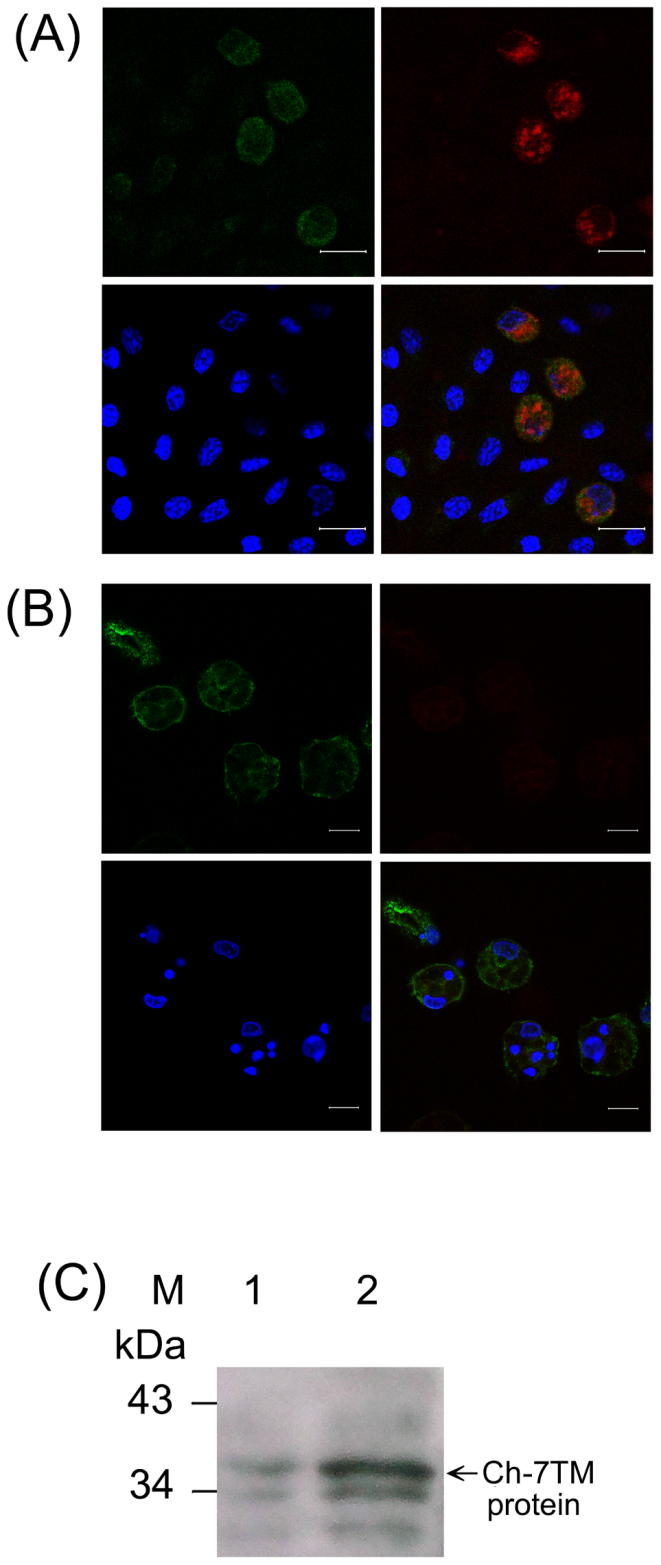
Determination of the Ch-7TM protein expressed in the adherent cells/1 h and 48 h of chicken PBMC. MAbs B28D5 and KUL01 were used in immunofluorescent double staining (A and B) and western blotting (C). Cells were grown on coverslips and fixed in cold acetone for 20 min. The adherent cells/1 h (A) and adherent cells/48 h (B) were visualized with PE-conjugated KUL01 antibody (right top), and subsequently double-stained with the Ch-7TM protein, using MAb B28D5 and Alexa 488-conjugated rabbit anti-mouse IgG as the primary and secondary antibodies, respectively (right bottom). DAPI was used for nuclear counterstaining (left bottom), and visualization was conducted as described in Fig. 3C. Bar: 10 µm. (C) Western blot analysis of the Ch-7TM protein. The PBMC was cultured for 1 h; the adherent cells were scraped and used as the adherent cells/1 h. Some of the adherent cells were cultured for an additional 48 h and treated as the adherent cells/48 h. The adherent cells/48 h (Lane 1) and adherent cells/1 h (Lane 2) were prepared for SDS-PAGE, and the proteins were transferred onto the PVDF membrane. The 1∶1×10^4^ dilution of B28D5 and 1∶5×10^5^ dilution of HRP-coupled goat anti-mouse Ig (Thermo Scientific, Pierce Biotechnology) were used as the primary and the secondary antibodies, respectively. The signals were enhanced using the SuperSignal® West Femto Maximum Sensitivity Substrate kit (Thermo Scientific). Molecular weights of the prestained standard markers (Fermentas) are indicated on the left.

To determine whether Ch-7TM protein expressed in the adherent cells of chicken PBMC, we also performed double staining experiments by using MAb KUL01 and MAb B28D5. The results demonstrated that the majority of permeabilized adherent cells/1 h were recognized with B28D5 (Fig. 4A, left top). The fact that some of B28D5 positive cells were double stained with KUL01 (Fig. 4A, right bottom) suggested that one subset of the adherent cells/1 h expressing Ch-7TM protein could likely be of the monocyte and macrophage lineage, as the KUL01 positive cells have been accepted to be the mononuclear phagocyte [18]. On the other hand, when the adherent cells were cultured for additional 48 h, nearly all adherent cells expressed Ch-7TM protein (Fig. 4B). However, the fluorescence intensity of KUL01 in the adherent cells/48 h decreased markedly compared with that of KUL01 in the adherent cells/1 h. It was reported that the intensity of KUL01 in macrophages decreased after activation through mechanisms such as avian influenza virus infection [19]. Our results were similar to the findings of their report, although the activation was due to additional 48 h culture of the adherent cells/1 h. The identity of the proteins recognized by B28D5 was verified. Western blot analysis indicated that a protein band with the expected size of 37.1 kDa reacted specifically with antibody B28D5 (Fig. 4C). The adherent cells/48 h displayed a weaker B28D5 signal intensity (Fig. 4C, lane 1) compared with the adherent cells/1 h prepared from the PBMC (Fig. 4C, lane 2), although some other small protein bands were also observed. These bands were probably generated as a result of the degradation of the Ch-7TM protein. Because the Ch-7TM gene encoded a novel protein, N-terminal sequencing [20] of the peptide fragments derived from the Ch-7TM protein digestion was required to confirm that the Ch-7TM proteins originated from the Ch-7TM gene. To achieve this goal, adequate amounts of this protein are required. However, this was difficult to achieve because only small amounts of the Ch-7TM protein were synthesized, because the Ch-7TM protein only could be detected using SuperSignal® West Femto Maximum Sensitivity substrate in western blot assays.

## Conclusions

We cloned a complete 7TM gene (Ch-7TM gene) by using the total RNA extracted from chicken adherent cells/48 h. We also cloned and characterized the Du-7TM- and Go-7TM-encoding regions which corresponded to the Ch-7TM-encoding region. An alignment of the sequences of the nucleotides and amino acids of the Ch-7TM-, Du-7TM-, and Go-7TM-encoding regions with those of the 7TM of the mallard predicted that the 7TMs of chicken, duck, and goose were the GPCR belonging to Family A of the 7TMs [6]. Double-staining with KUL01 and B28D5 MAbs suggested that the Ch-7TM receptors were expressed in subsets of the adherent cells of chicken PBMC, among which a subset that was doubly recognized with both MAbs was likely of the monocyte and macrophage lineage. The fluorescence intensities of B28D5 and, particularly, KUL01, decreased markedly when the adherent cells were cultured for additional 48 h.

The 7TM receptors comprise a large versatile family of membrane proteins, which mediate a vast array of cell signaling and signal transduction processes [5]. Given its surface location, particular motifs of the Ch-7TM protein are likely to be exposed to the chicken environment, making this receptor an attractive candidate for interacting with extracellular factors. Expression of the Ch-7TM receptor may mediate many biological activities. To further investigate the role of the Ch-7TM receptor, we developed a MAb B28D5 against Ch-7TM proteins. We are currently using MAb combined with siRNA technology to determine what the biological functions of the Ch-7TM receptor may be if the Ch-7TM mRNA is blocked.
